# Differential expression of inhibitory receptor NKG2A distinguishes disease‐specific exhausted CD8^+^ T cells

**DOI:** 10.1002/mco2.111

**Published:** 2022-01-10

**Authors:** Xiangyu Chen, Yao Lin, Shuai Yue, Yang Yang, Xinxin Wang, Zhiwei Pan, Xiaofan Yang, Leiqiong Gao, Jing Zhou, Zhirong Li, Li Hu, Jianfang Tang, Qing Wu, Yifei Wang, Qin Tian, Yaxing Hao, Lifan Xu, Bo Zhu, Qizhao Huang, Lilin Ye

**Affiliations:** ^1^ School of Laboratory Medicine and Biotechnology Southern Medical University Guangzhou China; ^2^ Institute of Cancer, Xinqiao Hospital Third Military Medical University Chongqing China; ^3^ Institute of Immunology Third Military Medical University Chongqing China; ^4^ Dermatology Hospital Southern Medical University Guangzhou China

**Keywords:** cancer immunotherapy, chronic viral infection, NKG2A, T cell exhaustion

## Abstract

Exhausted CD8^+^ T (Tex) cells are caused by persistent antigenic stimulation during chronic viral infection or tumorigenesis. Tex cells upregulate and sustain the expressions of multiple immune inhibitory receptors (IRs). Blocking IRs of Tex cells, exemplified by PD‐1, can partially restore their effector functions and thus lead to viral suppression or tumor remission. Tex cells derived from chronic viral infections share the expression spectrum of IRs with Tex cells derived from tumors; however, whether any IRs are selectively expressed by tumor‐derived Tex cells or virus‐derived Tex cells remains to be learnt. In the study, we found that Tex cells upregulate IR natural killer cell lectin‐like receptor isoform A (NKG2A) specifically in the context of tumor but not chronic viral infection. Moreover, the NKG2A expression is attributed to tumor antigen recognition and thus bias expressed by tumor‐specific Tex cells in the tumor microenvironment instead of their counterparts in the periphery. Such dichotomous NKG2A expression further dictates the differential responsiveness of Tex cells to NKG2A immune checkpoint blockade. Therefore, our study highlighted NKG2A as a disease‐dependent IR and provided novel insights into the distinct regulatory mechanisms underlying T cell exhaustion between tumor and chronic viral infection.

## Graphical abstract

In the study, we profiled the inhibitory receptors (IRs) of exhausted CD8^+^ T (Tex) cells recognizing the same epitope but developed in distinct diseases. We found Tex cells derived from tumor show remarkably higher NKG2A expression than Tex cells derived from chronic viral infection. Blocking NKG2A augments the progenitor to transitory differentiation of tumor Tex cells, accompanied by reinforced effector function and increased proliferation.

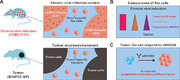

## 1 INTRODUCTION

In contrast to effector to memory CD8^+^ T cell differentiation followed by antigen clearance in acute viral infection, persistent antigen stimulation in chronic viral infection and tumor seeds the generation of exhausted CD8^+^ T (Tex) cells.^1^, ^2^ Accumulated evidences suggested that Tex cells in both diseases share core features of T cell exhaustion, including impaired effector functions, reduced proliferative capacities, lost memory potential, as well as elevated and sustained expression of an array of inhibitory receptors (IRs) that dampen T cell receptor signaling as compared to effector and memory CD8^+^ T cells.^1^, ^2^

The tremendous importance of IRs is demonstrated by the discovery of the role of programmed cell death (PD)‐1 in T cell exhaustion during chronic viral infections^3^, ^4^ and tumors,^5^, ^6^ thus driving PD‐1‐directed immunotherapy for cancer patients. Indeed, monoclonal antibodies (mAbs) against PD‐1 or its ligand PD ligand 1 (PD‐L1) have been approved in several indications, including metastatic melanoma, non‐small cell lung cancer, bladder cancer, kidney cancer, and Hodgkin lymphoma.^7^, ^8^ In addition, therapeutic trials or applications are also advanced for other IRs, including cytotoxic T lymphocyte antigen (CTLA)‐4,^9^ lymphocyte activation gene (LAG)‐3,^10^ T cell immunoglobulin and mucin domain‐containing (Tim)‐3,^11^, ^12^ and T cell immunoreceptor with immunoglobulin and immunoreceptor tyrosine‐based inhibition motif (ITIM) domains (TIGIT).^13^ Recently, the NK cell receptor NKG2A was highlighted as a novel IR expressed by Tex cells and blockade of this molecule resulted in durable tumor control.^14^, ^15^

However, the clinical advances of IRs‐targeted immunotherapies in chronic viral infections seem to be not matched for tumors. For instance, only modest effects were observed in hepatitis C virus (HCV)‐infected^16^ or human immunodeficiency virus (HIV)‐infected^17^ patients who received anti‐PD‐1 therapies. With hindsight, chronic viral infection and tumor are two different types of diseases with distinct physiopathology.^18^ Despite intensive studies in T cell exhaustion, very little is known about the differences of T cell exhaustion between these two distinct diseases in regard to IRs. Herein, we aimed to investigate the potential IRs that are expressed by Tex cells for a given epitope in a disease‐dependent manner. Furthermore, the mechanisms underlying the modulation of the disease‐specific IR(s) and Tex cell response upon blockade of the disease‐specific IR(s) were also explored.

## 2 RESULTS

### 2.1 Profiling the IRs of disease‐specific T cell exhaustion

**FIGURE 1:**
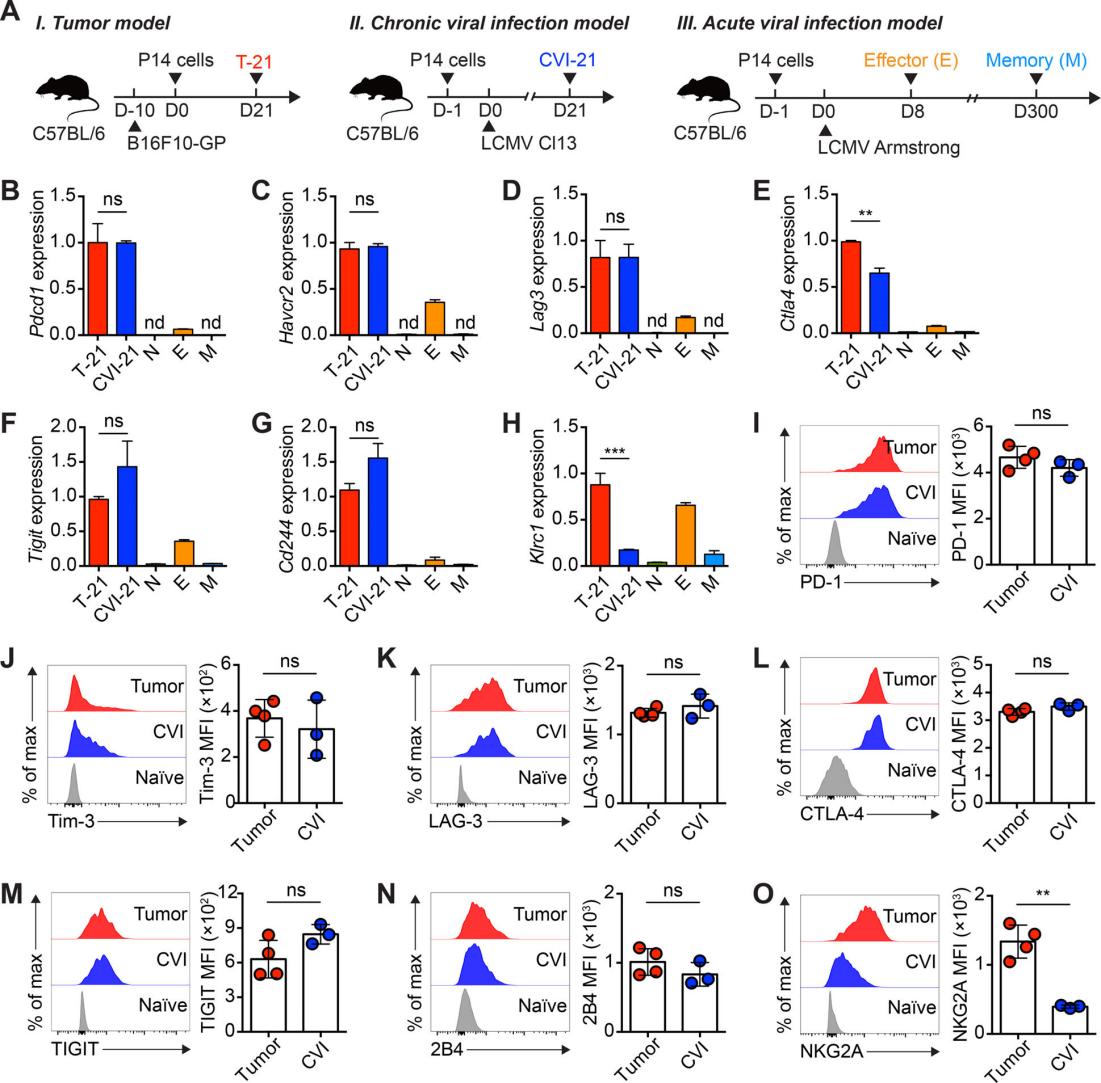
Comparisons of multiple inhibitory receptors in Tex cells from tumor and chronic viral infection. (A) Experimental schemes. (B‐H) Relative expressions of *Pdcd1* (B), *Havcr2* (C), *Lag3* (D), *Ctla4* (E), *Tigit* (F), *Cd244* (G), and *Klrc1* (H) among tumor (T)‐21, chronic viral infection (CVI)‐21, naïve (N), effector (E), and memory (M) P14 CD8^+^ T cells in (A). (I‐O) Flow cytometry analysis of PD‐1 (I), Tim‐3 (J), LAG‐3 (K), CTLA‐4 (L), TIGIT (M), 2B4 (N), and NKG2A (O) protein levels among T‐21, CVI‐21, naïve (N), effector (E), and memory (M) P14 CD8^+^ T cells in (A). Mean fluorescence intensity, MFI. The data are representative of two independent experiments. nd, not detected; ns, not significant. **p < 0.01; ****p < 0.0001. Error bars in (B‐O) indicate standard deviation (SD)

To probe the possible disease‐specific IR(s) of Tex cells, we sought to compare Tex cells that originated from the same antigen‐specific naïve CD8^+^ T cells but developed in either tumor microenvironment or chronic viral infection. To this end, congenic naïve (CD45.1^+^CD44^lo^CD62L^hi^) P14 CD8^+^ T cells, which specifically recognize lymphocytic choriomeningitis virus (LCMV) glycoprotein (GP) epitope H‐2D^b^ GP_33‐41_ epitope, were adoptively transferred into C57BL/6 recipients (CD45.2^+^) engrafted with B16F10 cells expressing the LCMV GP (hereafter referred to as B16F10‐GP cells) or C57BL/6 recipients (CD45.2^+^) that were subsequently infected with LCMV Clone 13 (Cl13) to set up a chronic viral infection (Figure 1A). In this scenario, we were able to compare the IRs of Tex cells recognizing the same epitope but developed in distinct diseases. As a control, we also transferred the same naïve P14 cells into recipients that were subsequently challenged with LCMV Armstrong to establish an acute viral infection (Figure 1A).

Firstly, P14 CD8^+^ T cells from the tumor tissues (B16F10‐GP day 21, T‐21), the spleens of chronic viral infection (LCMV Cl13 day 21, CVI‐21), or the spleens of acute viral infection (LCMV Armstrong day 8, effector; LCMV Armstrong day 300, memory) were sorted at indicated time points, and RNAs from these cells were subsequently extracted for measuring the transcripts of an array of IRs by quantitative real time‐polymerase chain reaction (qRT‐PCR) (Figure 1A and Figure S1). Consistent with previous studies,^1^, ^2^, ^18^, ^19^ the transcripts encoding PD‐1 (*Pdcd1*), Tim‐3 (*Havcr2*), LAG‐3 (*Lag3*), CTLA‐4 (*Clta4*), TIGIT (*Tigit*), and 2B4 (*Cd244*) were exclusively expressed by Tex cells of tumor and chronic viral infection as compared to those of the naïve, effector, and memory cells (Figure 1B‐G). Besides, the abundances of these transcripts were comparable between Tex cells of tumor and their counterparts of chronic viral infection (Figure 1B‐G). Remarkably, we observed that the transcript of NKG2A (*Klrc1*) was significantly more highly expressed in Tex cells of tumor than that in Tex cells of chronic viral infection (Figure 1H). Furthermore, we also found no disease preference of IR proteins, including PD‐1, Tim‐3, LAG‐3, CTLA‐4, TIGIT, and 2B4, in Tex cells by flow cytometry (Figure 1I‐N). By contrast, the NKG2A protein was noticeably expressed by Tex cells of tumor but not chronic viral infection (Figure 1O), echoing its disease preference at transcript level (Figure 1H). Taken together, these results suggest a shared expression profile of most IRs in Tex cells of tumor and chronic viral infection, except a preference of NKG2A expression in tumor‐specific Tex cells.

### 2.2 The NKG2A/CD94 heterodimer is highly expressed by Tex cells of tumor but not chronic viral infection

**FIGURE 2:**
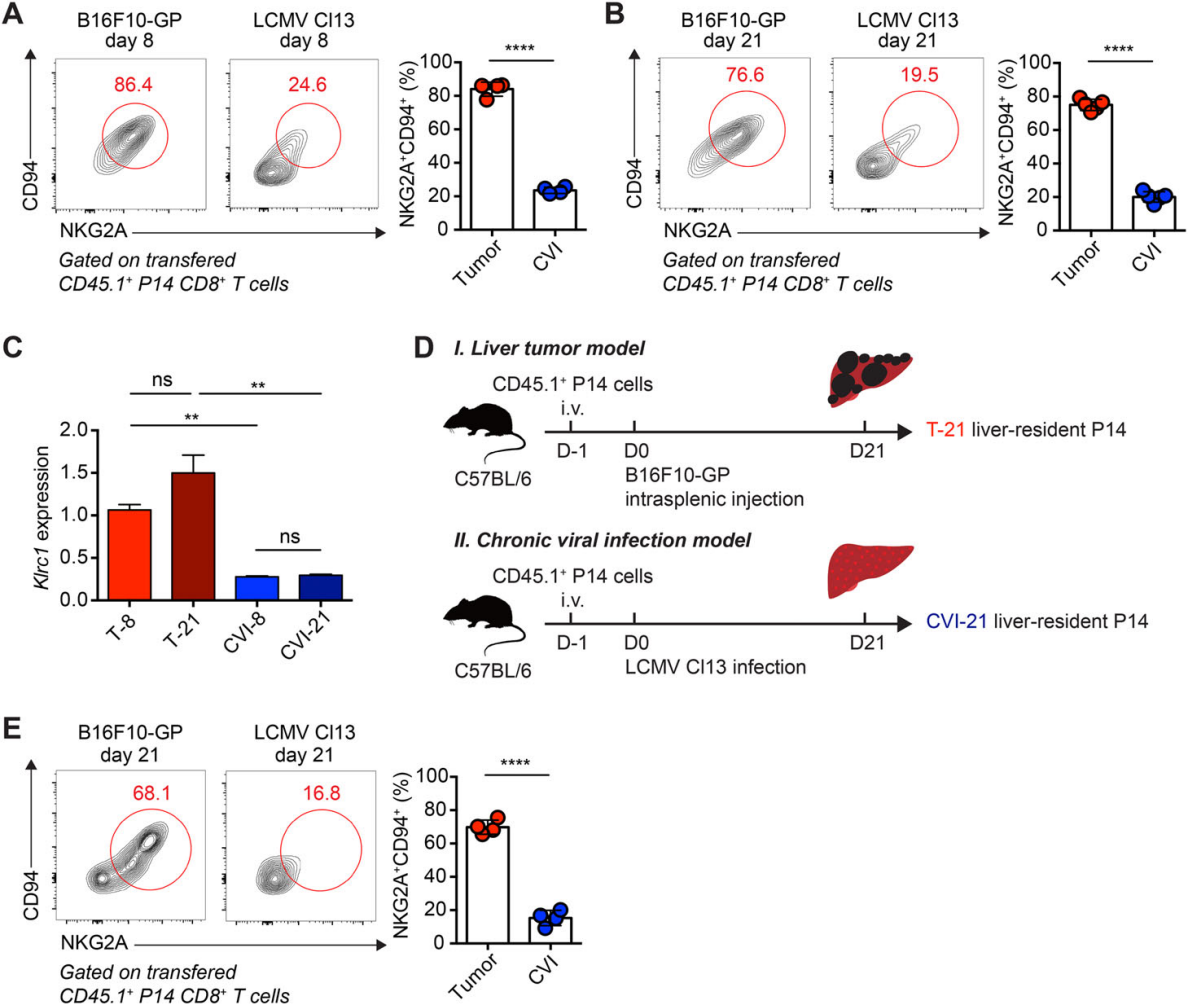
Expression bias of NKG2A in Tex cells from tumor but not chronic viral infection. (A and B) Flow cytometry analysis of transferred CD45.1^+^ P14 CD8^+^ T cells from the subcutaneous tumor tissues of B16F10‐glycoprotein (GP)‐engrafted recipients or the spleens of lymphocytic choriomeningitis virus (LCMV) Cl13‐infected recipients on day 8 (A) or day 21 (B) after P14 cell transfer. The numbers adjacent to the outlined areas indicate the percentages of NKG2A^+^CD94^+^ P14 cells. (C) Relative expression of *Klrc1* in T‐8, T‐21, CVI‐8, and CVI‐21 P14 cells. (D) Experimental scheme. (E) Flow cytometry analysis of transferred CD45.1^+^ P14 CD8^+^ T cells in (D). The numbers adjacent to the outlined areas indicate the percentages of NKG2A^+^CD94^+^ P14 cells. The data are representative of two independent experiments. ns, not significant. **p < 0.01; ****p < 0.0001. Error bars in (A‐C, E) indicate SD

Next, we longitudinally investigated the expression of NKG2A and its co‐receptor CD94 on disease‐specific Tex cells. In the early stage of T cell exhaustion (day 8), the NKG2A/CD94 co‐expression was found on up to 80% of tumor‐specific Tex cells but was much less observed (∼20%) in the counterparts of chronic viral infection (Figure 2A). This expression preference of NKG2A/CD94 heterodimeric assembly was also applied to tumor‐specific Tex cells in the late stage of T cell exhaustion (day 21) (Figure 2B). Consistently, greater amounts of the NKG2A transcript (*Klrc1*) were also observed in tumor‐specific Tex cells on both day 8 and day 21 as compared to these in chronic viral infection‐specific Tex cells (Figure 2C). In the scenario of acute viral infection, the NKG2A/CD94 co‐expression was found on ∼50% of effector P14 cells on day 8 postinfection and ended up with less than 10% in memory P14 cells on day 300 postinfection (Figure S2). These results indicate a continuous enrichment of NKG2A/CD94 heterodimer in Tex cells originating from tumor microenvironment but not chronic viral infection.

To avoid potential confounding tissue‐specific NKG2A/CD94 expression signatures, we further transferred congenic naïve P14 T cells (CD45.1^+^) into recipients (CD45.2^+^) that were subsequently infected with LCMV Cl13 or engrafted with B16F10‐GP cells intrasplenically to launch chronic viral infection or melanoma metastasis in the liver tissues (Figure 2D). On day 21 of each disease, we found NKG2A/CD94 heterodimer was generally expressed by a majority of liver tumor‐specific Tex cells (∼70%) but selectively expressed by liver Tex cells of chronic viral infection (∼15%) (Figure 2E), indicating that the disease type primarily discriminates NKG2A/CD94 expression of Tex cells.

In addition to the transferred P14 cells, we observed that more than 90% of endogenous H‐2D^b^ GP_33‐41_‐tetramer stained tumor‐infiltrating CD8^+^ T cells were NKG2A‐postive (Figure S3A,B). Furthermore, we also analyzed ovalbumin (OVA)‐specific OT1 cells, which specifically recognize the H‐2K^b^ OVA_257–264_ epitope, in recipient mice engrafted with B16F10 cells expressing OVA (hereafter referred to as B16F10‐OVA) (Figure S3C). As expected, we found a significant abundance of NKG2A protein in transferred OT1 cells under tumor microenvironment (Figure S3D,E). Thus, these findings highlight NKG2A as an IR preferentially expressed by Tex cells in tumor microenvironment rather than chronic viral infection.

### 2.3 Recognition of tumor antigens contributes to the differentiation of NKG2A/CD94^+^ Tex cells

**FIGURE 3:**
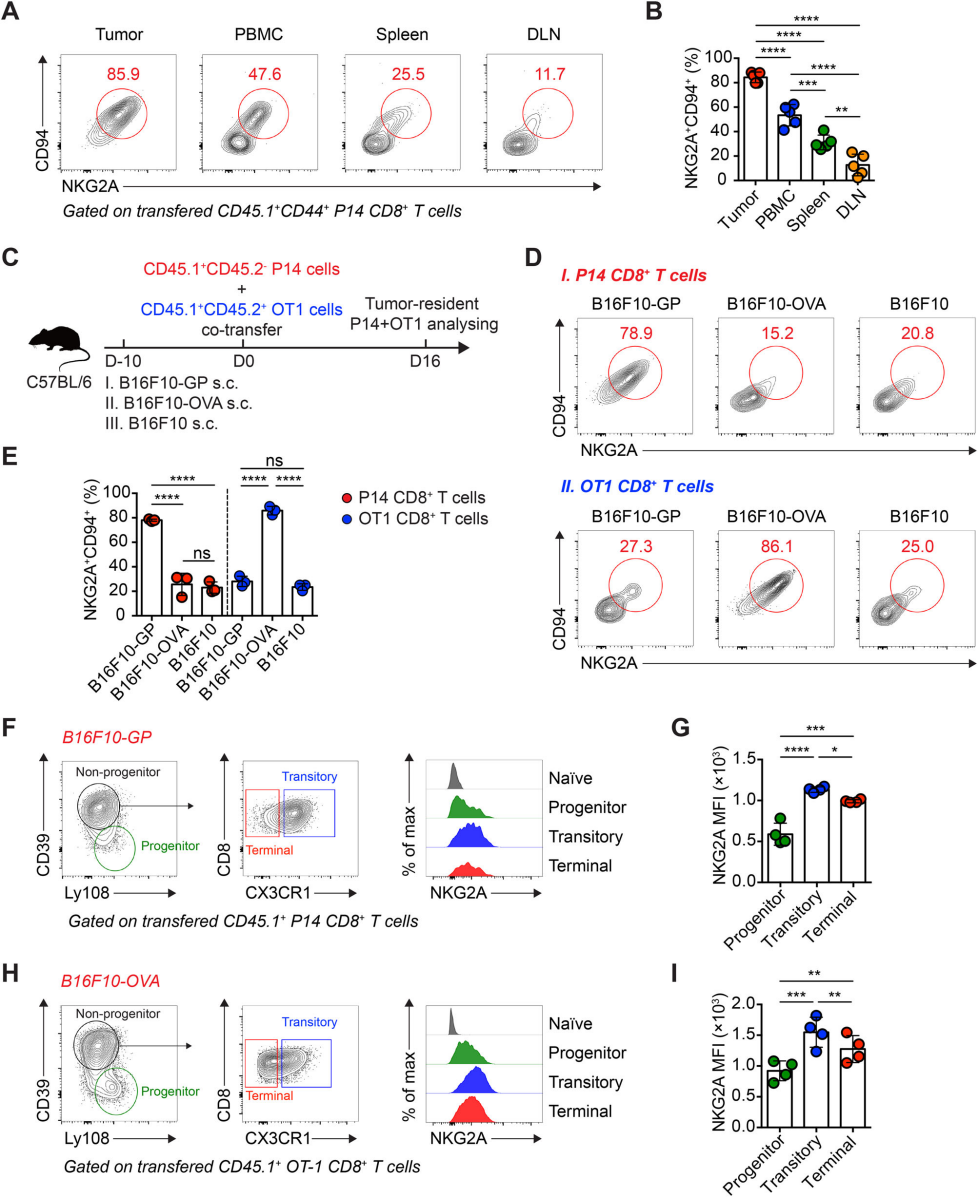
Tumor antigen abundance is closely related to NKG2A expression in tumor‐specific Tex cells. (A) Flow cytometry analysis of transferred CD45.1^+^ P14 CD8^+^ T cells from the subcutaneous tumor tissue, peripheral blood mononuclear cells (PBMCs), spleen, and DLN of B16F10‐glycoprotein (GP)‐engrafted recipients on day 21 after P14 cell transfer. The numbers adjacent to the outlined areas indicate the percentages of NKG2A^+^CD94^+^ P14 cells, which are summarized in (B). (C) Experimental scheme. (D) Flow cytometry analysis of transferred CD45.1^+^CD45.2^−^ P14 CD8^+^ T cells and CD45.1^+^CD45.2^+^ OT1 CD8^+^ T cells in (C). The numbers adjacent to the outlined areas indicate the percentages of NKG2A^+^CD94^+^ P14 cells or OT1 cells, which are summarized in (E). (F) Flow cytometry analysis of transferred CD45.1^+^ P14 CD8^+^ T cells from the subcutaneous tumor tissue of B16F10‐GP‐engrafted recipients on day 21 after P14 cell transfer. The left and middle panels indicate gating strategy of progenitor, transitory, and terminal Tex cells. The right panel shows the mean fluorescence intensity (MFI) of NKG2A in each Tex subsets, which are summarized in (G). (H) Flow cytometry analysis of transferred CD45.1^+^ OT1 CD8^+^ T cells from the subcutaneous tumor tissue of B16F10‐ovalbumin (OVA)‐engrafted recipients on day 21 after OT1 cell transfer. The left and middle panels indicate gating strategy of progenitor, transitory and terminal Tex cells. The right panel shows the MFI of NKG2A in each Tex subsets, which are summarized in (I). The data are representative of two independent experiments. ns, not significant. *p < 0.05; **p < 0.01; ***p < 0.001; ****p < 0.0001. Error bars in (B, E, G, and I) indicate SD

NKG2A expression is upregulated by chronic antigenic stimulation.^20^, ^21^ To identify whether high amounts of tumor antigens contribute to the robust NKG2A/CD94 expression in tumor‐specific Tex cells, we firstly delineate the NKG2A/CD94^+^ tumor‐specific P14 cells in the tumor and peripheral lymphoid tissues of B16F10‐GP‐engrafted recipients. As anticipated, the NKG2A/CD94 expression of P14 cells in the peripheral lymphoid tissues, including peripheral blood mononuclear cells (PBMCs), the spleen, and tumor draining lymphoid node (DLN), was much less pronounced than that in the tumor (Figure 3A,B). This phenomenon suggests that NKG2A/CD94 receptors are more restricted to tumor‐specific CD8^+^ T cells in the tumor microenvironment rather than their counterparts in the periphery.

To further confirm the role of tumor antigens in driving NKG2A/CD94 expression of tumor‐specific CD8^+^ T cells, anti‐CD3/anti‐CD28‐activated P14 cells (CD45.1^+^CD45.2^−^) and OT1 cells (CD45.1^+^CD45.2^+^) were co‐transferred into C57BL/6 recipients (CD45.1^−^CD45.2^+^) engrafted with B16F10‐GP or B16F10‐OVA or B16F10 (Figure 3C). On day 16 postcell transfer, we noticed that P14 cells preferentially upregulate NKG2A/CD94 expression in B16F10‐GP tumor rather than B16F10‐OVA tumor or B16F10 tumor, whereas OT1 cells preferentially upregulate NKG2A/CD94 expression in B16F10‐OVA tumor rather than B16F10‐GP tumor or B16F10 tumor (Figure 3D,E). Thus, these findings indicate that high amounts of tumor antigens elicit the NKG2A/CD94 expression of tumor‐specific CD8^+^ T cells.

Tex cells are heterogeneous and encompassing various cellular subsets at distinct differentiation, including TCF‐1^hi^Ly108^hi^CD39^lo^ progenitor, CD39^hi^CX3CR1^hi^ transitory, and CD39^hi^CX3CR1^lo^ terminal Tex cells. Progenitor Tex cells give rise to transitory Tex cells and subsequently to terminal Tex cells, while transitory and terminal Tex cells elicit effector function.^22^, ^23^, ^24^, ^25^, ^26^ Low amounts of antigens drive the differentiation of progenitor Tex, while high amounts of antigens promote the differentiation of transitory and terminal Texs.^27^ Indeed, we found that the highest level of NKG2A expression in transitory Tex compared to those in progenitor Tex and terminal Tex, and terminal Tex expressed a relatively higher level of NKG2A than that of progenitor Tex (Figure 3F‐I). Together, these observations support the notion that NKG2A expression in tumor‐specific Tex cells is largely attributed to the abundant tumor antigens within tumor microenvironment.

The major histocompatibility complex (MHC) complex class Ib protein, Qa‐1^b^, serves as the ligand presenting peptide antigens to murine NKG2A/CD94 receptors, and the engagement transduces inhibitory signal to effector cells.^28^, ^29^, ^30^, ^31^ To explore whether disease‐specific Qa‐1^b^ expression pattern is involved in modulating Tex cell function via NKG2A/CD94 receptors, we detected the Qa‐1^b^ protein in major cell types under the conditions of tumor microenvironment and chronic viral infection. Of all detected cell types, we noticed an abundant Qa‐1^b^ expression in tumor cells as compared to dendritic cells, macrophages, and myeloid‐derived suppressor cells from both tumor and chronic viral infection (Figure S4). Collectively, abundant tumor antigens might culminate in the generation of Tex cells with abundant NKG2A/CD94 heterodimer, which serve as receptors for Qa‐1^b^ on tumor cells to transduce inhibitory signals to the Tex cells.

### 2.4 NKG2A blockade bolsters proliferation and effector function of tumor‐specific Tex cells

**FIGURE 4:**
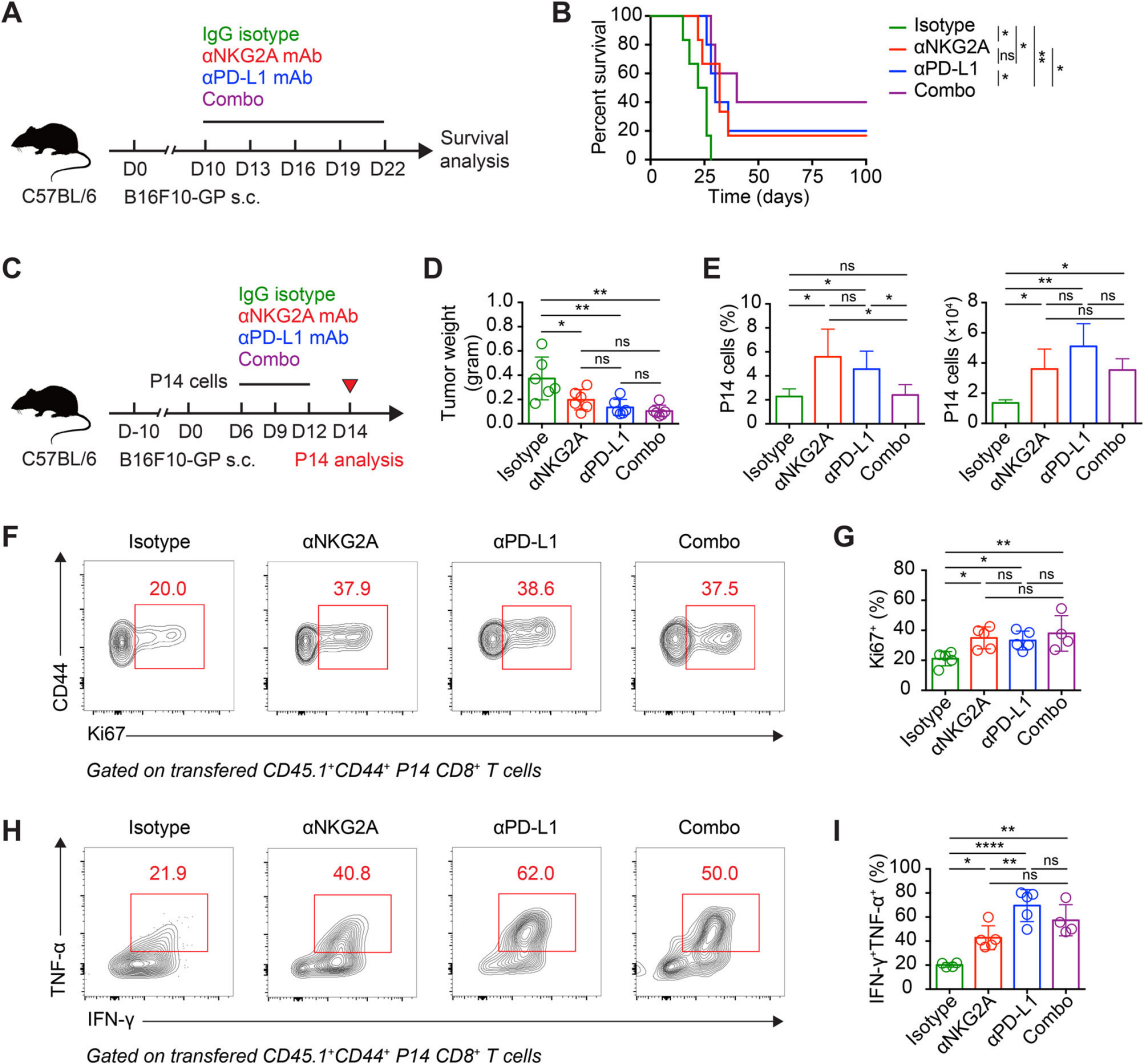
NKG2A blockade augments tumor‐specific Tex cell response. (A) Experimental scheme. (B) Survival curves of C57BL/6 mice in (A). (C) Experimental set‐up. (D) Tumor weight of C57BL/6 mice sacrificed on day 14 described in (C). (E) Frequencies (left) and numbers (right) of tumor‐infiltrating P14 CD8^+^ T cells. (F) Flow cytometry analysis of transferred CD45.1^+^ P14 CD8^+^ T cells from the tumor tissues of B16F10‐glycoprotein (GP)‐engrafted recipients on day 14 after P14 cell transfer as described in (C). The numbers adjacent to the outlined areas indicate the percentages of Ki67^+^ P14 cells, which are summarized in (G). (H) Flow cytometry analysis of transferred CD45.1^+^ P14 CD8^+^ T cells from the tumor tissues of B16F10‐GP‐engrafted recipients on day 14 after P14 cell transfer as described in (C). The numbers adjacent to the outlined areas indicate the percentages of IFN‐γ^+^TNF‐α^+^ P14 cells, which are summarized in (I). The data are representative of two independent experiments. ns, not significant. *p < 0.05, **p < 0.01; ****p < 0.0001. Error bars in (D, E, G, and I) indicate SD

We then aimed to assess the effects of NKG2A blockade on tumor‐specific Tex cells and resultant anti‐tumor response. To this end, C57BL/6 mice engrafted with B16F10‐GP cells were administrated with anti‐NKG2A‐blocking mAb or control IgG mAb on day 10, 13, 16, 19, and 22. As positive controls, B16F10‐GP cells‐engrafted mice were treated with anti‐PD‐L1‐blocking mAb or anti‐NKG2A/anti‐PD‐L1‐blocking mAbs (Figure 4A). We found that ∼20% of B16F10‐GP tumor recipients who received anti‐NKG2A‐blocking mAb or anti‐PD‐L1‐blocking mAb were in complete remission, while the recipients of control group were not able to survive at day 25 posttumor engraftment (Figure 4B). Remarkably, a synergistic effect of anti‐NKG2A/anti‐PD‐L1‐blocking mAbs rescued ∼40% recipients from death (Figure 4B).

To further investigate how tumor‐specific Tex cells respond to NKG2A blockade, congenically marked CD45.1^+^ P14 cells were transferred into B16F10‐GP cell‐engrafted C57BL/6 recipients, which were subsequently administrated with anti‐NKG2A‐blocking mAb or anti‐PD‐L1‐blocking mAb or anti‐NKG2A/anti‐PD‐L1‐blocking mAbs or control mAb on day 6, 9, and 12 (Figure 4C). All the mice were sacrificed on day 14, and the weight of excised tumors were determined. Again, we observed a significant reduction of tumor weight in groups with NKG2A blockade or PD‐L1 blockade or NKG2A/PD‐L1 blockade (Figure 4D). Within the tumor, the magnitude of P14 cell response was largely reinforced by NKG2A blockade or PD‐L1 blockade or NKG2A/PD‐L1 blockade as compared to that by isotype mAb (Figure 4E). Consistently, P14 cells of NKG2A blockade, PD‐L1 blockade, and NKG2A/PD‐L1 blockade showed increased proliferation, as reflected by Ki67 staining (Figure 4F,G). Notably, effector functions of P14 cells, as indicated by the capacity of producing effector cytokines interferon (IFN)‐γ and tumor necrosis factor (TNF)‐α, were augmented by NKG2A blockade, PD‐L1 blockade, and NKG2A/PD‐L1 blockade (Figure 4H,I). However, these NKG2A blockade‐induced beneficial features were not observed in P14 cells originating from the tumor DLN (Figure S5), coinciding with the less observed NKG2A expression of tumor‐specific CD8^+^ T cells in DLN (Figure 3A). Therefore, blocking NKG2A promotes the proliferation and effector function of tumor‐infiltrating Tex cells, the magnitude of which resembles that fostered by PD‐L1 blockade to some extents.

### 2.5 NKG2A blockade fosters the differentiation of transitory Tex cells in tumor microenvironment

**FIGURE 5:**
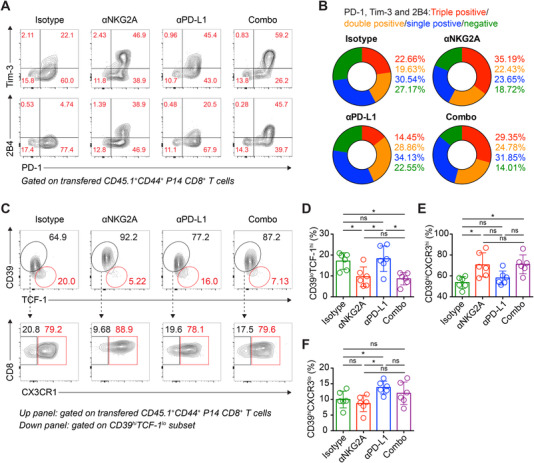
NKG2A blockade accelerates the progenitor to transitory differentiation of tumor‐specific Tex cells. (A) Flow cytometry analysis of PD‐1, Tim‐3, and 2B4 expression in transferred CD45.1^+^ P14 CD8^+^ T cells from the tumor tissues of B16F10‐glycoprotein (GP)‐engrafted recipients on day 14 after P14 cell transfer. These recipients received NKG2A blockade or PD‐L1 blockade or NKG2A/PD‐L1 blockade or control mAb as described in Figure 4C. (B) Pie charts presenting proportions of triple positive (red), double positive (orange), single positive (blue), and negative (green) of PD‐1, Tim‐3, and 2B4 expressions in P14 cells in (A). (C) Flow cytometry analysis of progenitor (CD39^lo^TCF‐1^hi^), transitory (CD39^hi^CX3CR1^hi^), and terminal (CD39^hi^CX3CR1^lo^) Tex cell subsets in tumor‐infiltrating P14 cells in (A), the frequencies of which are summarized in (D), (E), and (F), respectively. The data are representative of two independent experiments. ns, not significant. *p < 0.05. Error bars in (D, E, and F) indicate SD

It is well acknowledged that progenitor Tex cells are featured by depressed cytolytic activities but reserved proliferative potential, while transitory/terminal Tex cells are featured by terminal differentiation, including accumulated IRs, increased cytolytic activities, and reduced proliferative potential.^22^, ^23^, ^24^, ^25^, ^26^ We next sought to ascertain the Tex differentiation in the scenario of NKG2A blockade. To do so, we firstly mapped cardinal IRs, including PD‐1, Tim‐3, and 2B4, in tumor‐specific Tex cells aforementioned (Figure 4C). As indicated, PD‐1/Tim‐3/2B4 triple‐positive subset is more distributed to Tex cells upon NKG2A blockade or NKG2A/PD‐L1 blockade than these upon PD‐L1 blockade or control mAb (Figure 5A,B), suggesting a terminally differentiated feature in Tex cells modified by NKG2A blockade. More detailed, NKG2A blockade or NKG2A/PD‐L1 blockade fostered less progenitor Tex cells but more transitory Tex cells as compared to these of PD‐L1 blockade or control mAb (Figure 5C‐F), recalling the abundant NKG2A expression in transitory Tex cells (Figure 3F‐I). Hence, these results demonstrate that NKG2A blockade might accelerate the progenitor to transitory differentiation of tumor‐specific Tex cells.

### 2.6 NKG2A blockade minimally affects Tex cell response in chronic viral infection

**FIGURE 6:**
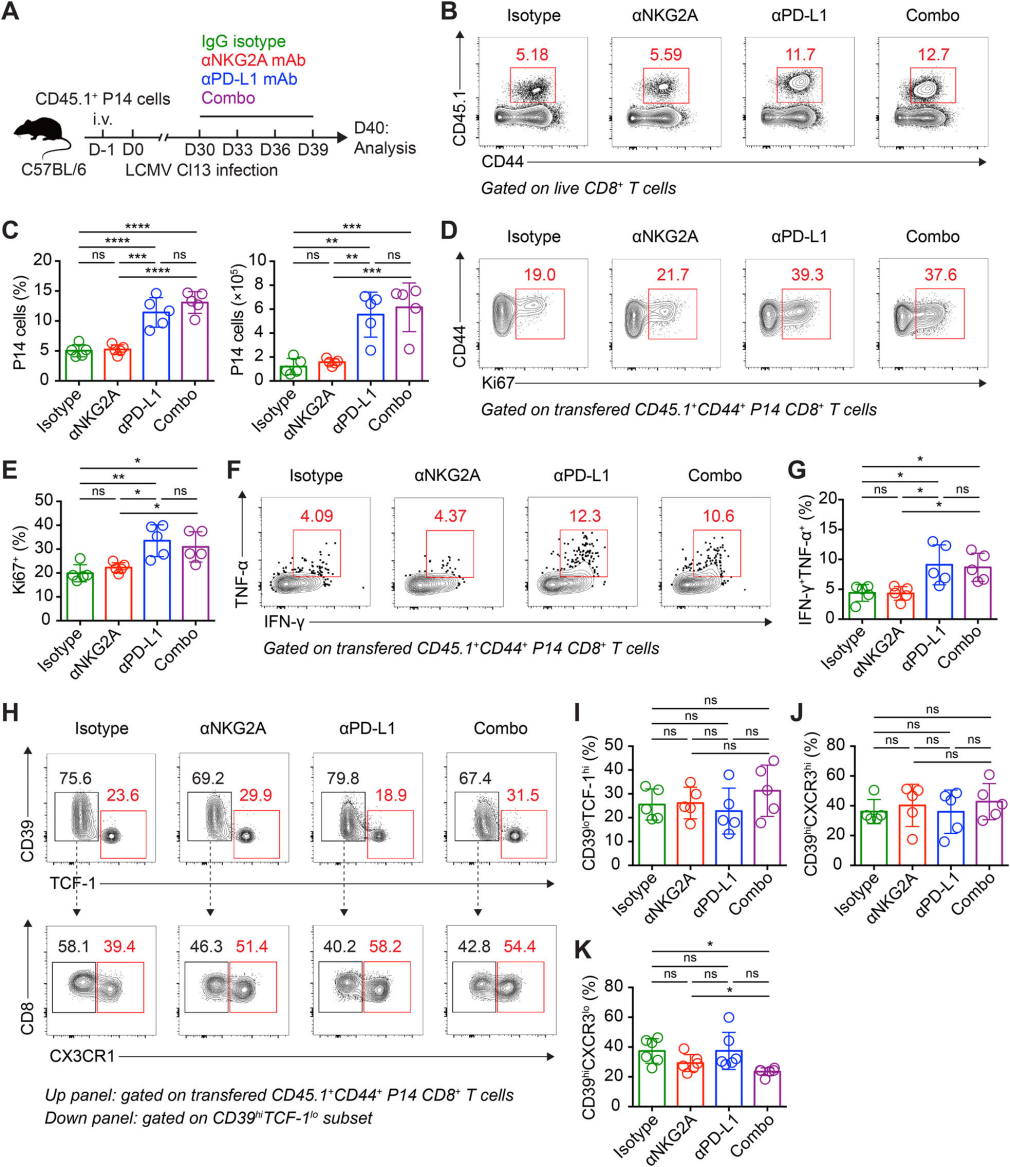
Tex cell response is minimally modified by NKG2A blockade during chronic viral infection. (A) Experimental scheme. (B) Flow cytometry analysis of transferred CD45.1^+^ P14 CD8^+^ T cells from the spleens of lymphocytic choriomeningitis virus (LCMV) Cl13‐infected recipients on day 40 postinfection as described in (A). The numbers adjacent to the outlined areas indicate the percentages of P14 cells. (C) The frequencies and numbers of transferred CD45.1^+^ P14 CD8^+^ T cells in (B). (D) Flow cytometry analysis of transferred CD45.1^+^ P14 CD8^+^ T cells from the spleens of LCMV Cl13‐infected recipients on day 40 postinfection as described in (A). The numbers adjacent to the outlined areas indicate the percentages of Ki67^+^ P14 cells, which are summarized in (E). (F) Flow cytometry analysis of transferred CD45.1^+^ P14 CD8^+^ T cells from the spleens of LCMV Cl13‐infected recipients on day 40 postinfection as described in (A). The numbers adjacent to the outlined areas indicate the percentages of IFN‐γ^+^TNF‐α^+^ P14 cells, which are summarized in (G). (H) Flow cytometry analysis of progenitor (CD39^lo^TCF‐1^hi^), transitory (CD39^hi^CX3CR1^hi^), and terminal (CD39^hi^CX3CR1^lo^) Tex cell subsets in tumor‐infiltrating P14 cells in (B), the frequencies of which are summarized in (I), (J), and (K), respectively. The data are representative of two independent experiments. ns, not significant. *p < 0.05. Error bars in (C, E, G, I, J, and K) indicate SD

It is of particular interest to explore whether NKG2A blockade also boosts Tex cell response in chronic viral infection, albeit the less pronounced NKG2A expression in these cells. For the purpose, congenic CD45.1^+^ P14 cells were transferred into C57BL/6 recipients, which were subsequently infected with LCMV Cl13 virus and administrated with anti‐NKG2A‐blocking mAb or anti‐PD‐L1‐blocking mAb, or anti‐NKG2A/anti‐PD‐L1‐blocking mAbs or control mAb on day 30, 33, 36, and 39 after infection (Figure 6A). Interestingly, both frequency and number of splenic P14 cells were comparable between the NKG2A blockade group and the control mAb group on day 40 postinfection (Figure 6B,C). Besides, anti‐NKG2A‐blocking mAb showed no synergic effects with anti‐PD‐L1‐blocking mAb in further reinforcing the P14 cell response (Figure 6B,C). Indeed, cell proliferation, effector function, and Tex cell differentiation of P14 cells were limitedly influenced by NKG2A blockade (Figure 6D‐K). Therefore, these findings suggest that Tex cell response is limitedly modified by NKG2A blockade in the context of chronic viral infection.

## 3 DISCUSSION

In the study, we found an expression bias of IR NKG2A in Tex cells from tumor but not chronic viral infection. The upregulation of NKG2A expression in tumor‐specific CD8^+^ T cells is closely related to tumor antigen abundance. Furthermore, blockade of NKG2A specifically bolsters proliferation, effector function, and terminal differentiation of Tex cells in tumor rather than their counterparts in chronic viral infection.

Chronic antigenic stimulation is reported as a crucial factor in driving NKG2A expression in CD8^+^ T cells.^20^, ^21^ Given the fact that Tex cells in chronic viral infection also receive persistent T cell receptor (TCR) signaling^2^ but show much lower NKG2A expression compared to that of Tex cells in tumor, signals specifically derived from the tumor microenvironment might also be required to induce and sustain the NKG2A expression in tumor‐specific Tex cells. The transforming growth factor beta (TGF‐β) is of paramount importance for modifying the tumor microenvironment.^32^, ^33^ Besides, TGF‐β is also involving in reinforcing NKG2A expression of in vitro cultured CD8^+^ T cells in a concentration‐dependent manner.^20^ Thus, factors exemplified by TGF‐β in tumor microenvironment might contribute to the abundant NKG2A/CD94 heterodimer in tumor‐specific Tex cells. Such factors warrant further investigation.

The NKG2A receptor contains ITIM and emits inhibitory signals via tyrosine phosphatase Src‐homology 2 domain phosphatase‐1 (SHP‐1).^34^ The high abundance of NKG2A expression is restricted to tumor‐specific CD8^+^ T cells in the tumor microenvironment rather than peripheral tumor‐specific CD8^+^ T cells or peripheral virus‐specific CD8^+^ T cells or tumor‐infiltrating bystander CD8^+^ T cells, thus endowing the NKG2A blockade an advantage of specifically promoting antigen‐specific CD8^+^ T cell response within the tumor. Also, no abnormalities have been observed in the mice devoid of CD94, the co‐receptor of NKG2A.^35^ Thus, blocking NKG2A might be a safe strategy in treating solid tumors.

Previous studies indicated that blockade of PD‐1/PD‐L1 pathway accelerates the differentiation of stem‐like progenitor Tex cells to effector‐like transitory Tex cells in chronic viral infection^22^, ^23^, ^24^ and tumor,^26^ thus curtailing viral infection and delaying tumor progression. Herein, we found that NKG2A blockade specifically mobilizes progenitor to transitory Tex cell differentiation in tumor and shows synergetic effects with PD‐L1 blockade in controlling tumorigenesis. However, no addictive effect was observed in the effector evaluation of tumor‐derived Tex cells; furthermore, Tex cell proportion was even lower upon combination of PD‐L1 blockade and NKG2A blockade than that upon PD‐L1 blockade or NKG2A blockade (Figure 4E). The mechanism underlying this discrepancy might be due to overactivation of Tex cells in the absence of both PD‐1 and NKG2A. Therefore, an optimal blockade therapy regimen combining PD‐1/PD‐L1 and NKG2A should be further investigated.

NKG2A receptor is also highly expressed by NK cells originated from tumors.^14^, ^15^ The durable tumor control induced by NKG2A blockade was abolished upon NK cell depletion,^14^ which suggests that NK cells also contribute to NKG2A blockade‐mediated anti‐tumor response and thus provides another explanation for the improved tumor control but no ulterior Tex cell response upon NKG2A blockade plus PD‐L1 blockade observed in the study. In addition, NK cells upregulate NKG2A expression during chronic viral infections.^36^, ^37^ Anti‐viral therapies result in a reduction of NKG2A expression in NK cells during hepatitis B virus (HBV) infection^36^ and HCV infection.^37^ Moreover, NKG2A blockade augments the antiviral activities of NK cells and curtails viral titers in HBV infection.^36^ In our study, we found that virus‐specific CD8^+^ T cells express moderate NKG2A and NKG2A blockade limitedly enhance the anti‐viral activities of Tex cell in a mouse model of chronic viral infection, which is consistent with the fact that simian immunodeficiency virus (SIV)‐specific CD8^+^ T cells lack NKG2A expression.^38^ However, we do not exclude the potential anti‐viral effects of NK cells upon NKG2A blockade in our chronic viral infection model, which needs further exploration.

In conclusion, we demonstrated that IR NKG2A is differentially expressed by Tex cell with a given TCR established during tumor and chronic viral infection development, which further dictates the differential responses of disease‐specific Tex cells to NKG2A blockade. To our knowledge, the present study provides the first concept of significant expression bias of IR(s) in Tex cells programmed in tumor and chronic viral infection, which highlights the importance of developing disease‐specific immunotherapy to respectively treat tumor and chronic viral infection. This disease‐biased expression of IRs, including but not limited to NKG2A, should be further investigated in the scope of human diseases.

## 4 MATERIALS AND METHODS

### 4.1 Mice, viruses, and cell lines

C57BL/6, OTI transgenic mice (expressing a TCR specific for the H‐2K^b^ OVA_257‐264_ epitope), and CD45.1^+^ congenic mice (strain B6.SJL‐*PtprcaPepcb*/BoyJ) were purchased from the Jackson Laboratories. P14 transgenic mice (carrying a transgenic T cell antigen receptor that recognizes H‐2D^b^ GP_33–41_ epitope) were gifted from Dr. Rafi Ahmed (Emory University). All the mice used in the study were analyzed at 6–10 weeks of age, and both genders were included without randomization or ‘‘blinding.’’

The LCMV Armstrong and Cl13 strains were provided by Dr. Rafi Ahmed (Emory University) and propagated in our laboratory. Mice were intraperitoneally infected with 2 × 10^5^ plaque‐forming units (PFU) LCMV Armstrong or intravenously with 2 × 10^6^ PFU LCMV Cl13 virus.

B16F10 cells and B16F10 cells expressing ovalbumin (OVA) (hereafter referred to as B16F10‐OVA) were purchased from ATCC. The B16F10 cells expressing GP of LCMV Cl13 strain was generated by CRISPR/Cas9‐mediated insertion of LCMV Cl13 GP gene sequence into the genome of B16F10 cells (herein referred as B16F10‐GP) and further selected by puromycin. All tumor cell lines were grown in complete DMEM‐10 medium: DMEM (Gibco), 10% FBS (Gibco), 1% penicillin/streptomycin (Gibco), and 1% L‐glutamine (Gibco). For B16F10‐GP cells, additional 100 U/ml puromycin is supplemented. For subcutaneous tumor models, mice were subcutaneously implanted with 5 × 10^5^ B16F10 or B16F10‐GP or B16F10‐OVA cells. Tumors were measured every 2 days to estimate the tumor size in two dimensions with a caliper. The tumor volume was calculated according to the formula (length × width^2^)/2. For the development of liver metastasis model, mice were injected intrasplenically with 3 × 10^5^ B16F10‐GP cells, followed by splenectomy 5 min after injection.^39^

### 4.2 Adoptive transfer of T cells

For LCMV virus infection models, 5 × 10^4^ (for LCMV Armstrong) or 2000 (for LCMV Cl13) congenically marked naïve splenic P14 cells were adoptively transferred into recipient mice 1 day before infection. For subcutaneous tumor models, tumor‐engrafted recipient mice were first intraperitoneally administrated with 4‐mg cyclophosphamide (CTX, Sigma, PHR1404) at 9 days after tumor implantation when the tumor is palpable, then 5 × 10^5^ congenic naïve splenic P14 cells or OT1 cells were adoptively transferred into these recipients. For liver metastasis model, congenic recipients were administrated with 4‐mg CTX and then transferred with 5 × 10^5^ naïve splenic P14 cells prior to the development of liver metastasis.

### 4.3 Lymphocytes isolation

Lymphocytes of the spleen and lymph node were obtained by mashing the spleen or lymph node through a 70‐μm nylon cell strainer (BD Falcon). The lymphocytes of PBMCs were obtained using FICOLL (TBD, LTS1077‐1) density gradient. To obtain tumor‐infiltrating lymphocytes (TILs), tumors were dissected from euthanized mice and mechanically minced. Then, TILs were enriched by using Percoll (GE Healthcare, 17‐0891‐09) density gradient. To harvest liver‐resident lymphocytes, livers were dissected, perfused with phosphate buffer saline (PBS), and mechanically minced. Then, liver‐resident lymphocytes were obtained using Percoll density gradient.

### 4.4 Flow cytometry

Antibodies were purchased from Biolegend or BD Phamingen and included CD8 (Biolegend, clone 53–6.7), CD44 (Biolegend, clone IM7), CD45.1 (Biolegend, clone A20), CD45.2 (Biolegend, clone 104), PD‐1 (Biolegend, RMP1‐30), Tim‐3 (Biolegend, B8.2C12), LAG‐3 (Biolegend, clone C9B7W), CTLA‐4, TIGIT (Biolegend, clone 4D4/mTIGIT), 2B4 (Biolegend, clone m2B4 (B6)458.1), NKG2A (Biolegend, clone 16A11), CD94 (Biolegend, clone 18d3), CD39 (Biolegend, Duha59), Ly108 (Biolegend, clone 330‐AJ), CX3CR1 (Biolegend, clone SA011F11), Qa‐1^b^ (BD, clone 6A8.6F10.1A6), F4/80 (Biolegend, clone BM8), CD11b (Biolegend, clone M1/70), CD11c (Biolegend, clone N418), MHC‐II (Biolegend, clone AF6‐120.1), Ly6C (BD, clone AL‐21), Ly6G (BD, clone 1A8), IFN‐γ (Biolegend, clone XMG1.2), and TNF‐α (Biolegend, clone MP6‐XT22). MHC‐I H‐2D^b^ GP_33‐41_‐tetramer was gifted from Dr. Rafi Ahmed (Emory University). Single cell suspensions were stained with antibodies for surface antigens and live/dead dye (Life Technologies) in PBS containing 2% FBS (wt/vol) on ice for 30 min. Intracellular staining for Ki67 (BD, 556026) and TCF‐1 (CST, 2206) was performed using the Foxp3 fixation/permeabilization kit (eBioscience, 00–5523). Intracellular staining for IFN‐γ and TNF‐α was performed by using cytofix/cytoperm kit (BD, 554714) according to manufacturer's instruction following a 5‐h in vitro restimulation with 0.2 g/ml LCMV GP_33‐41_ peptide (for peripheral lymphocytes) or phorbol myristate acetate/ionmycin (for TILs) in the presence of brefeldin A and monensin. Cells were collected on a Canto II flow cytometer (BD), and data were analyzed using FlowJo software (Tree Star). All the contour plots containing more than 1000 events were shown. Sorting was performed on a FACSAria III (BD).

### 4.5 qRT‐PCR

Transferred CD45.1^+^ P14 CD8^+^ T cells were sorted from the spleens or tumors of recipient mice following a gating strategy of CD8^+^CD44^+^CD45.1^+^CD45.2^−^Lin^−^Live/Dead dye^−^. Total RNA of sorted P14 cells was extracted from the sorted cells with a Micro Total RNA Isolation Kit (Thermo Fisher, AM1931) and reverse‐transcribed using RevertAid Minus First Strand cDNA Synthesis Kit (Thermo Scientific, K1632). Total RNA of the spleen or tumor tissue was extracted using TRIzol LS (Life Technologies) and reverse‐transcribed using RevertAid Minus First Strand cDNA Synthesis Kit. The relative expression levels of transcripts were determined using AceQ qPCR SYBR Green Master Mix (Vazyme, Q111) on a CFX96 Touch Real‐Time System (Bio‐Rad). The primers used in the study: *Pdcd1* (forward: 5′‐ACCCTGGTCATTCACTTGGG‐3′; reverse: 5′‐CATTTGCTCCCTCTGACACTG‐3′), *Havcr2* (forward: 5′‐TCAGGTCTTACCCTCAACTGTG‐3′; reverse: 5′‐GGCATTCTTACCAACCTCAAACA‐3′), *Lag3* (forward: 5′‐CTGGGACTGCTTTGGGAAG‐3′; reverse: 5′‐GGTTGATGTTGCCAGATAACCC‐3′), *Ctla4* (forward: ; reverse:), *Tigit* (forward: 5′‐CCACAGCAGGCACGATAGATA‐3′; reverse: 5′‐CATGCCACCCCAGGTCAAC‐3′), *Cd244* (forward: 5′‐AGCCCTGGACTAATGGGACTT‐3′; reverse: 5′‐GCTGGCGTCAATCTGGTTCT‐3′), *Klrc1* (forward: 5′‐GCCCCTGCAAAGGTTTTCC‐3′; reverse: 5′‐TCTGTGGGTTCTAGTCATTGAGG‐3′), and *Hprt* (forward: 5′‐TCAGTCAACGGGGGACATAAA‐3′; reverse: 5′‐GGGGCTGTACTGCTTAACCAG ‐3′).

### 4.6 In vitro culture of CD8^+^ T cells

Splenic naïve (CD44^−^CD62L^+^) P14 or OT1 CD8^+^ T cells were sorted by flow cytometry and in vitro cultured with complete RPMI‐1640 medium containing 10% FBS, 1% penicillin/streptomycin, 0.1% β‐mercaptoethanol (Gibco), 10 ng/ml recombinant human interleukin (IL)‐2 (Proteintech) in the presence of 2 μg/ml anti‐CD3, and 0.5 μg/ml anti‐CD28 at 37°C for 72 h.

### 4.7 Antibody treatments

For immune‐checkpoint blockade experiments, tumor‐engrafted or LCMV Cl13‐infected mice were administrated with 200‐μg αNKG2A (BioXCell, BE0321, clone 20D5) or αPD‐L1 (BioXCell, BE0101, clone 10F.9G2) or isotype‐matched control antibody (BioXCell, rat IgG2b) via intraperitoneal injection at indicated time points.

### 4.8 Statistical analysis

Statistical analysis was performed with Prism 6.0 (GraphPad) software. For comparisons between two independent groups, significance was determined by two‐tailed unpaired Student's *t*‐test. For comparisons among more than two groups, one‐way ANOVA test was used to determine the significance. The log‐rank (Mantel‐Cox) test was performed for comparing survival curves among groups. *p* values less than 0.05 were defined as statistically significant. Asterisks were used to indicate significance correspond with: *p *< 0.05^*^, *p *< 0.01^**^, *p *< 0.001^***^, and *p *< 0.0001^****^.

## CONFLICTS OF INTEREST

The authors declare no conflict of interest.

## ETHICS APPROVAL

All mouse experiments were performed with the guidelines of the Institutional Animal Care and Use Committees of the Third Military Medical University. Mice were sacrificed at the indicated time points, or when the estimated tumor volume reached 2000 mm^3^.

## AUTHOR CONTRIBUTIONS

Xiangyu Chen and Lilin Ye conceived the study. Xiangyu Chen, Yao Lin, Shuai Yue, Yang Yang, Xinxin Wang, Zhiwei Pan, Xiaofan Yang, Leiqiong Gao, Jing Zhou, Zhirong Li, and Li Hu performed experiments. Jianfang Tang, Qing Wu, Yifei Wang, Qin Tian, Yaxing Hao, and Lifan Xu helped study designs and discussed the data. Xiangyu Chen analyzed the data and drafted the manuscript with Lilin Ye, Qizhao Huang, and Bo Zhu.

## Supporting information



Supporting Information

## Data Availability

All the data are available from the corresponding authors upon reasonable request.
