# Supplementary material for: Differential expression of inhibitory receptor NKG2A distinguishes disease‐specific exhausted CD8+ T cells
Source: MedComm (2020). 2022 Jan 10;3(1):e111. doi: 10.1002/mco2.111 (PMC8906559; doi:10.1002/mco2.111)
Supplement: Supplementary file 1 — Supporting Information [file MCO2-3-e111-s001.pdf]

Differential expression of inhibitory receptor NKG2A distinguishes disease-specific exhausted CD8$^+$ T cells

Running title: NKG2A is an IR specific for tumor Tex

Xiangyu Chen[1,2,5], Yao Lin[3,5], Shuai Yue[3,5], Yang Yang[1,5], Xinxin Wang[2], Zhiwei Pan[3], Xiaofan Yang[4], Leiqiong Gao[3], Jing Zhou[3], Zhirong Li[3], Li Hu[3], Jianfang Tang[3], Qing Wu[3], Yifei Wang[1], Qin Tian[4], Yaxing Hao[3], Lifan Xu[3], Bo Zhu[2,*], Qizhao Huang[1,*], Lilin Ye[1,3,*]

[1]School of Laboratory Medicine and Biotechnology, Southern Medical University, Guangzhou, 510515, China.

[2]Institute of Cancer, Xinqiao Hospital, Third Military Medical University, Chongqing, 400038, China.

[3]Institute of Immunology, Third Military Medical University, Chongqing, 400038, China.

[4]Dermatology Hospital, Southern Medical University, Guangzhou, 510091, China.

[5]These authors contributed equally to this work.

*Corresponding authors. *E-mail* addresses: bo.zhu@tmmu.edu.cn (B. Zhu), huangqizhao1988@163.com (Q. Huang) and yelilinlcmv@tmmu.edu.cn (L. Ye).

Figure S1

A Tumor model (B16F10-GP, day 21)

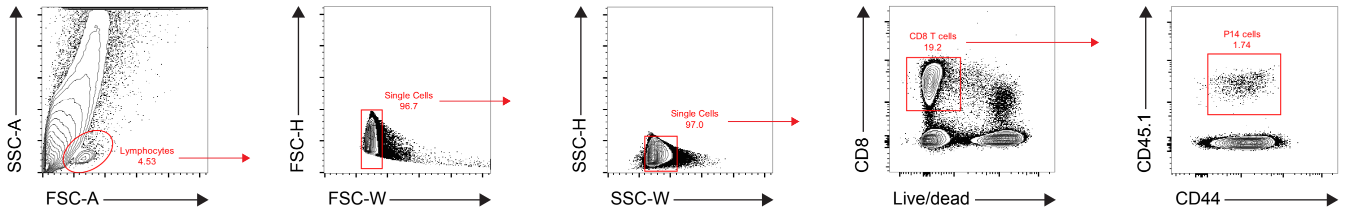

B Chronic viral infection model (LCMV Cl13, day 21)

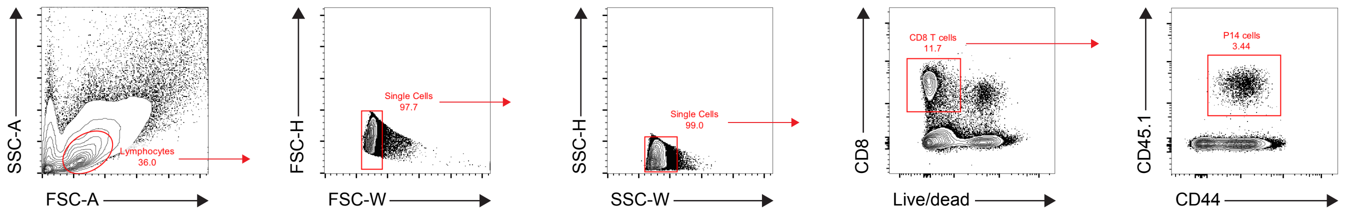

C Acute viral infection model (LCMV Armstrong, day 300)

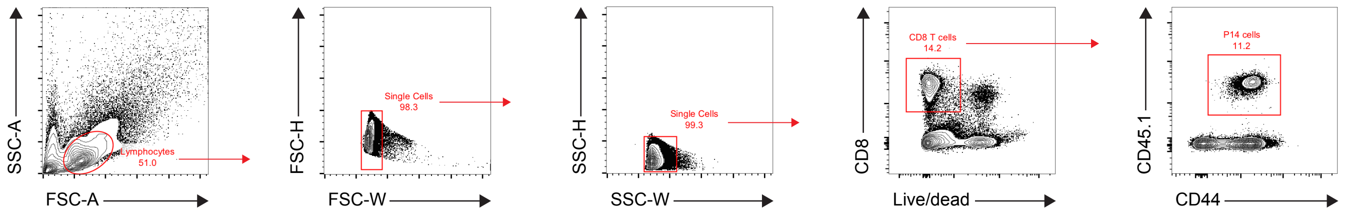

Figure S1. Gating strategies of transferred CD45.1$^+$ P14 CD8$^+$ T cells in different models. Flow cytometry analysis of transferred CD45.1$^+$ P14 CD8$^+$ T cells from the B16F10-GP tumors (A) or the spleens of LCMV Cl13-infected recipients (B) or the spleens of LCMV Armstrong-infected recipients (C).

Figure S2

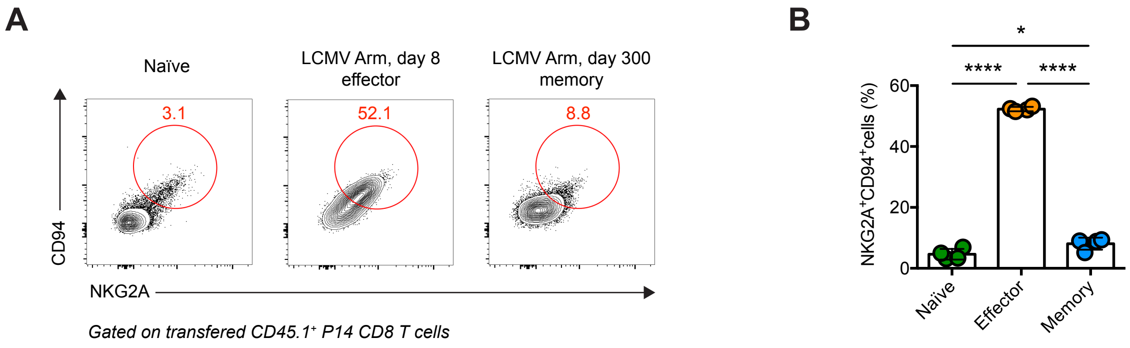
A
Naïve
LCMV Arm, day 8 effector
LCMV Arm, day 300 memory
3.1
52.1
8.8
CD94
NKG2A
Gated on transfered CD45.1+ P14 CD8 T cells
B
NKG2A+CD94+cells (%)
60
40
20
0
*
****
****
Naïve
Effector
Memory

Figure S2. NKG2A/CD94 expressions in naïve, effector and memory P14 cells during acute viral infection. (A) Flow cytometry analysis of transferred $CD45.1^+$ P14 $CD8^+$ T cells from the spleens of LCMV Armstrong-infected recipients on indicated time points or naïve P14 cells. The numbers adjacent to the outlined areas indicate the percentages of $NKG2A^+CD94^+$ P14 cells, which are summarized in (B). The data are representative of two independent experiments. *$p < 0.05$ and ****$p < 0.0001$. Error bars in (B) indicate SD.

Figure S3

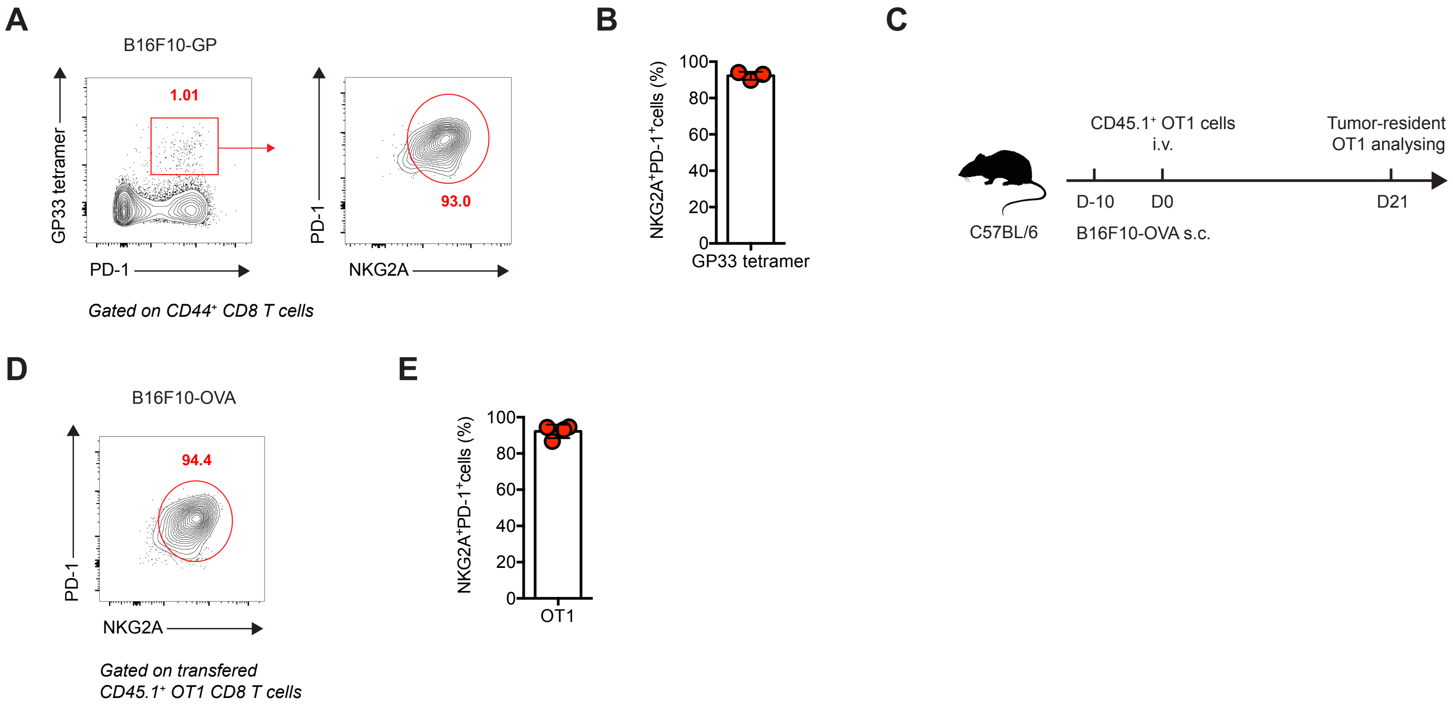
A
B16F10-GP
1.01
93.0
GP33 tetramer
PD-1
PD-1
NKG2A
Gated on CD44⁺ CD8 T cells
B
100
80
60
40
20
0
NKG2A⁺PD-1⁺cells (%)
GP33 tetramer
C
CD45.1⁺ OT1 cells
i.v.
Tumor-resident
OT1 analysing
D-10
D0
D21
C57BL/6
B16F10-OVA s.c.
D
B16F10-OVA
94.4
PD-1
NKG2A
Gated on transfered
CD45.1⁺ OT1 CD8 T cells
E
100
80
60
40
20
0
NKG2A⁺PD-1⁺cells (%)
OT1

Figure S3. NKG2A expression in endogenous H-$2D^b$ GP_{33-41}-$tetramer^+CD8^+$ T cells in B16F10-GP tumor and in transferred OT1 cells in B16F10-OVA tumor. (A) Flow cytometry analysis of $NKG2A^+$ subset in endogenous H-$2D^b$ GP_{33-41}-$tetramer^+CD8^+$ T cells from the tumors of B16F10-GP engrafted mice on day 21-post B16F10-GP cells engraftment. (B) The frequency of $NKG2A^+PD\text{-}1^+$ subset in H-$2D^b$ GP_{33-41}-$tetramer^+CD8^+$ T cells. (C) Experimental scheme. (D) Flow cytometry analysis of transferred $CD45.1^+$ OT1 $CD8^+$ T cells from the subcutaneous tumor tissues of B16F10-OVA engrafted recipients on day 21 after P14 cell transfer. The numbers adjacent to the outlined areas indicate the percentages of $NKG2A^+PD\text{-}1^+$ OT1 cells, which are summarized in (E). The data are representative of two independent experiments. Error bars in (B, E) indicate SD.

Figure S4

A

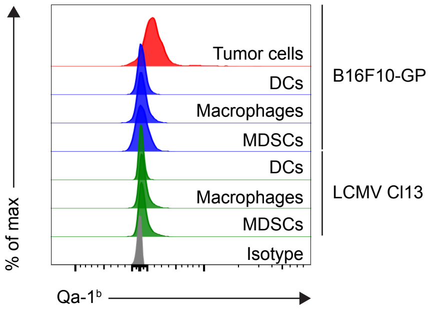
Tumor cells
DCs
Macrophages
MDSCs
DCs
Macrophages
MDSCs
Isotype
B16F10-GP
LCMV Cl13
% of max
Qa-1b

B

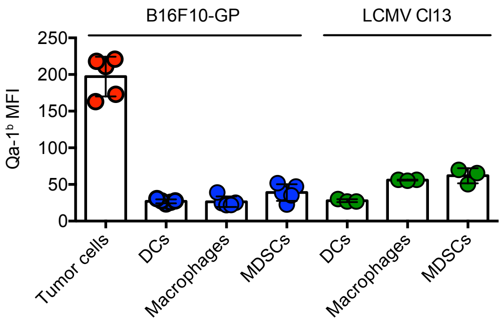
B16F10-GP
LCMV Cl13
Qa-1b MFI
250
200
150
100
50
0
Tumor cells
DCs
Macrophages
MDSCs
DCs
Macrophages
MDSCs

Figure S4. Qa-1^b expression of major cell types in tumor and chronic viral infection. (A) Flow cytometry analysis of Qa-1^b expression levels in indicated cell types from subcutaneous tumor tissues of B16F10-GP-engrafted mice or the spleens of LCMV Cl13-infected mice. (B) The MFI of Qa-1^b protein in indicated cell types. The data are representative of two independent experiments. Error bars in (B) indicate SD.

Figure S5

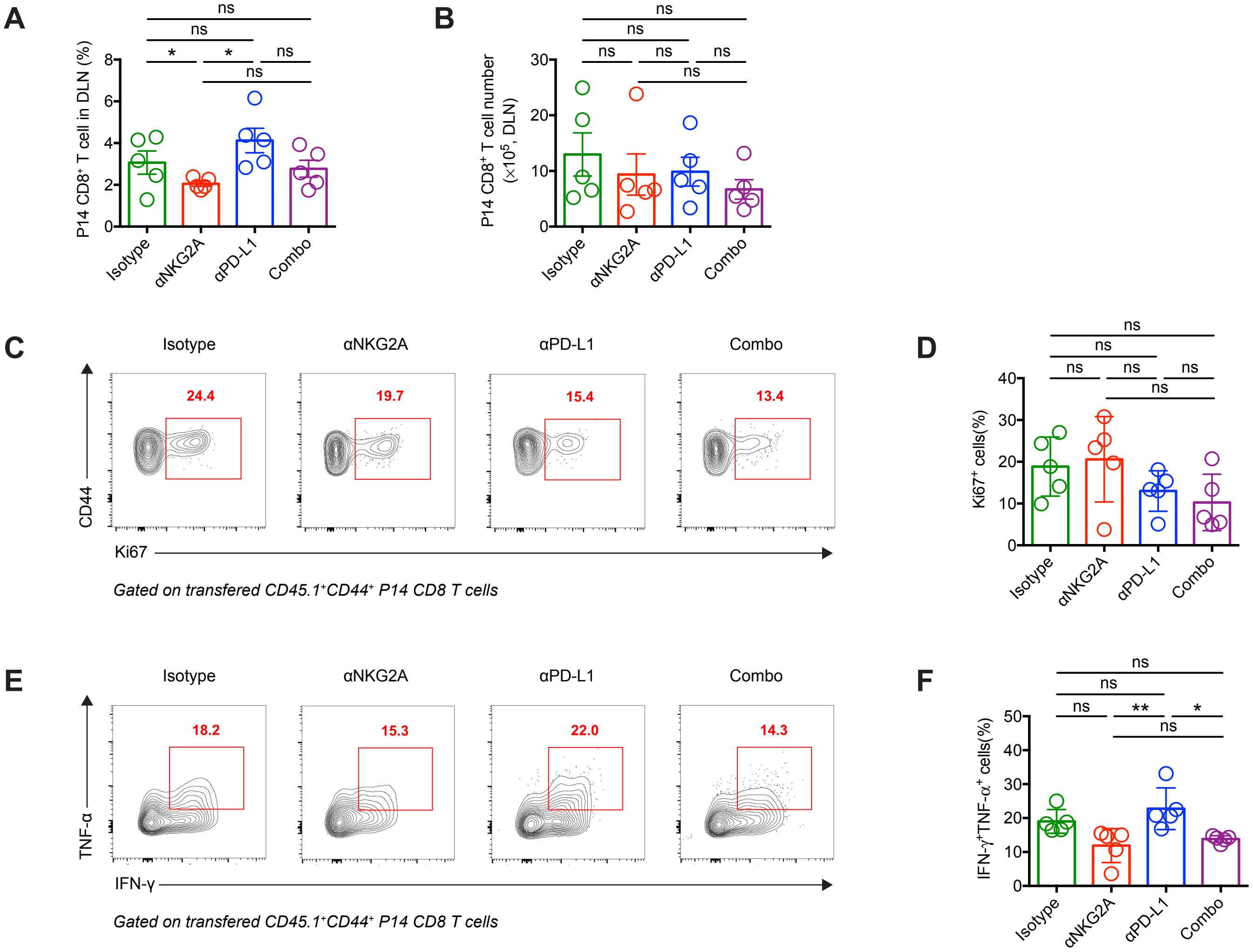

A
P14 CD8+ T cell in DLN (%)
Isotype
αNKG2A
αPD-L1
Combo
ns
*
B
P14 CD8+ T cell number (×10^5, DLN)
C
CD44
Ki67
24.4
19.7
15.4
13.4
Gated on transfered CD45.1+CD44+ P14 CD8 T cells
D
Ki67+ cells(%)
E
TNF-α
IFN-γ
18.2
15.3
22.0
14.3
F
IFN-γ+TNF-α+ cells(%)
**

Figure S5. NKG2A blockade limitedly affects tumor-specific $CD8^+$ T cells in tumor DLN. (A, B) Frequencies (A) and numbers (B) of tumor-DLN P14 $CD8^+$ T cells described in Figure 4C. (C) Flow cytometry analysis of transferred $CD45.1^+$ P14 $CD8^+$ T cells from the tumor DLN of B16F10-GP engrafted recipients in (A). The numbers adjacent to the outlined areas indicate the percentages of $Ki67^+$ P14 cells, which are summarized in (D). (E) Flow cytometry analysis of transferred $CD45.1^+$ P14 $CD8^+$ T cells from the tumor DLN in (A). The numbers adjacent to the outlined areas indicate the percentages of IFN-γ^+TNF-α^+ P14 cells, which are summarized in (F). The data are representative of two independent experiments. *$p < 0.05$ and **$p < 0.01$. Not significant, ns. Error bars in (A, B, D, F) indicate SD.
